# Elevated circulating levels of the interferon-γ–induced chemokines are associated with disease activity and cutaneous manifestations in adult-onset Still’s disease

**DOI:** 10.1038/srep46652

**Published:** 2017-04-24

**Authors:** Jae Ho Han, Chang-Hee Suh, Ju-Yang Jung, Mi-Hyun Ahn, Mi Hwa Han, Ji Eun Kwon, Hyunee Yim, Hyoun-Ah Kim

**Affiliations:** 1 Department of Pathology, Ajou University School of Medicine, 164 Worldcup-ro, Yeongtong-gu, Suwon 443-380, Korea; 2 Department of Rheumatology, Ajou University School of Medicine, 164 Worldcup-ro, Yeongtong-gu, Suwon 443-380, Korea

## Abstract

C-X-C motif chemokine 9 (CXCL9), CXCL10, and CXCL11 are produced in response to interferon-γ (IFN-γ) and trigger inflammation with the accumulation of activated lymphocytes. It appears that these chemokines could play a role in the pathogenesis of adult-onset Still’s disease (AOSD). Therefore, we investigated the associations between the levels of these chemokine and clinical manifestations in patients with active AOSD. Serum levels of IFN-γ, CXCL9, CXCL10 and CXCL11 were determined using enzyme-linked immunosorbent assays. IFN-γ levels were higher in AOSD patients than in rheumatoid arthritis (RA) patients (*p* = 0.001) or healthy controls (HCs) (*p* = 0.032). AOSD patients also exhibited higher levels of CXCL9, CXCL10, and CXCL11 compared with RA patients (*p* < 0.001) and HCs (*p* < 0.001). In follow-up AOSD patients after treatment with corticosteroid, the levels of CXCL9, CXCL10 and CXCL11 fell significantly, whereas IFN-γ levels were not significantly different. On immunohistochemistry, the percentage of CXCL10-positive inflammatory cells was higher in skin biopsy samples from AOSD patients than in those from normal control (*p* = 0.012), eczema (*p* = 0.019), and psoriasis (*p* = 0.009) groups. Levels of the IFN-γ–induced chemokines, CXCL9, CXCL10 and CXCL11, were elevated and correlated with several disease activity markers. These interferon-γ–induced chemokines may contribute to inflammatory responses and skin manifestations in AOSD.

Adult-onset Still’s disease (AOSD), a systemic inflammatory disorder of unknown etiology, is considered an adult form of systemic juvenile inflammatory arthritis (JIA). It is characterized by a spiking fever, evanescent skin rash, sore throat, myalgia, arthritis, and organomegaly^1^,^2^. Leukocytosis with neutrophilia, thrombocytosis, elevated liver enzymes and C-reactive protein (CRP), and hyperferritinemia are present in AOSD, although these results are non-specific. AOSD is a very rare disease, but the prognosis for some patients could be poor because of disease severity or complications, such as fulminant hepatitis, severe myocarditis, or macrophage activation syndrome^3^,^4^.

The etiology of AOSD is unknown, but immune dysfunction has been suggested to underlie disease development^1^,^5^. Proinflammatory cytokines, such as interleukin-1β (IL-1β), tumor necrosis factor-α (TNF- α), IL-6, CXCL8, and IL-18, are elevated in acute AOSD patients^6^,^7^. Although the role of interferon-γ (IFN-γ) in AOSD is controversial, signature IFN-γ–induced cytokines or chemokines, such as IL-18 or C-X-C motif chemokine 10 (CXCL10), are significantly elevated in AOSD^5^,^6^,^7^,^8^. Our previous study also showed that CXCL10 levels are elevated in AOSD patients compared with rheumatoid arthritis (RA) patients and healthy controls (HCs), and are correlated with AOSD disease activity markers^9^.

The CXC chemokines CXCL9 (monokine induced by IFN-γ [MIG]), CXCL10 (IFN-γ–induced protein-10 [IP-10]), and CXCL11 (IFN-induced T cell α chemoattractant [I-TAC]) bind CXCR3 (C-X-C motif receptor-3) and thus are collectively referred to as IFN-γ–inducible CXCR3 ligands^10^,^11^. They are also induced by specific Toll-like receptor ligands or cytokines, and are expressed by several cell types^12^,^13^,^14^. These CXCR3 ligands have been reported to mediate chemotactic and proliferative responses in collaboration or competition with each other^13^. Although CXCR3 ligands and their functions have been reported in autoimmune diseases, such as JIA and RA, relatively few studies have investigated the role of these chemokines in the pathogenesis of AOSD^8^,^9^,^15^,^16^,^17^.

The aim of the present study was to evaluate the role of IFN-γ and IFN-γ–induced chemokines in AOSD. To this end, we determined the serum levels of IFN-γ, CXCL9, CXCL10 and CXCL11, and investigated their associations with clinical disease activities and manifestations in patients with active, untreated AOSD. To explore the *in vivo* involvement of these chemokines in AOSD, we performed an immunohistochemical analysis of skin biopsies for CXCL9, CXCL10 and CXCL11, and their receptor, CXCR3, in 34 patients with active, untreated AOSD.

## Results

### Clinical characteristics of patients

Table 1 summarizes the clinical characteristics of the 39 AOSD patients, 30 RA patients, and 28 HCs. The mean age of AOSD patients was 42.9 ± 16.8 years, and 84.6% of patients were female. There were no significant differences in age or gender among groups. The principal clinical symptoms of AOSD patients included a high spiking fever (94.9%), skin rash (87.2%), arthritis (53.8%), sore throat (56.4%), and splenomegaly (20.5%). All AOSD patients were in the initial stages of high-level disease activity before treatment. Of the AOSD patients, only four (10.2%) were diagnosed with reactive hemophagocytic syndrome (RHS). These patients were diagnosed with RHS by based on clinical features or tissue biopsy^18^.

### Serum IFN-γ, CXCL9, CXCL10, and CXCL11 levels

**Figure 1:**
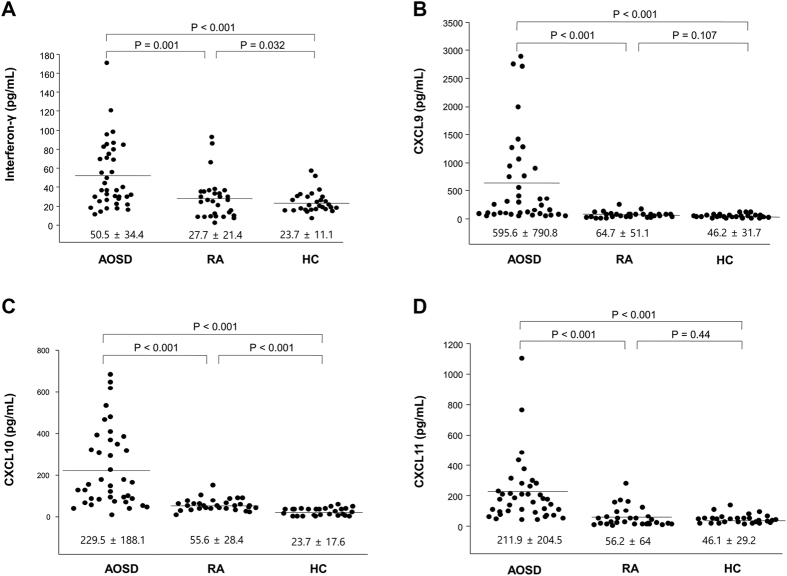
Levels of interferon-γ (IFN-γ) (**A**), CXC chemokine 9 (CXCL9) (**B**), CXCL10 (**C**) and CXCL11 (**D**) in 39 active, untreated adult-onset Still’ disease (AOSD) patients, 30 rheumatoid arthritis (RA) patients and 28 healthy controls (HCs). Data are expressed as means ± SDs and were analyzed using independent t-tests.

Figure 1 shows the IFN-γ, CXCL9, CXCL10 and CXCL11 levels in AOSD patients, RA patients, and HCs. IFN-γ levels in AOSD patients (50.5 ± 34.4 pg/mL) were higher than those in RA patients (27.7 ± 21.4 pg/mL; *p* = 0.001) and HCs (23.7 ± 11.1 pg/mL; *p* < 0.001). CXCL9 levels in AOSD patients (595.6 ± 790.8 pg/mL) were higher than those in RA patients (64.7 ± 51.1 pg/mL; *p* < 0.001) and HCs (46.2 ± 31.7 pg/mL; *p* < 0.001). CXCL10 levels in AOSD patients (229.5 ± 188.1 pg/mL) were higher than those in RA patients (55.6 ± 28.4 pg/mL; *p* < 0.001) and HCs (23.7 ± 17.6 pg/mL; *p* < 0.001). CXCL11 levels in AOSD patients (211.9 ± 204.5 pg/mL) were higher than those in RA patients (56.2 ± 64.0 pg/mL; *p* < 0.001) and HCs (46.1 ± 29.2 pg/mL; *p* < 0.001).

A comparison of IFN-γ, CXCL9, CXCL10, and CXCL11 levels showed that serum levels of CXC chemokines were significantly higher among AOSD patients with RHS than in patients without RHS (CXCL9: 1622.5 ± 867.6 vs. 478.2 ± 703.1 pg/mL, *p* = 0.004; CXCL10: 494.0 ± 119.6 vs. 199.2 ± 170.6 pg/mL, *p* = 0.003; CXCL11: 513.0 ± 412.1 vs. 177.5 ± 140.9 pg/mL, *p = *0.017). However, IFN-γ levels were not significantly different between the two groups.

**Figure 2:**
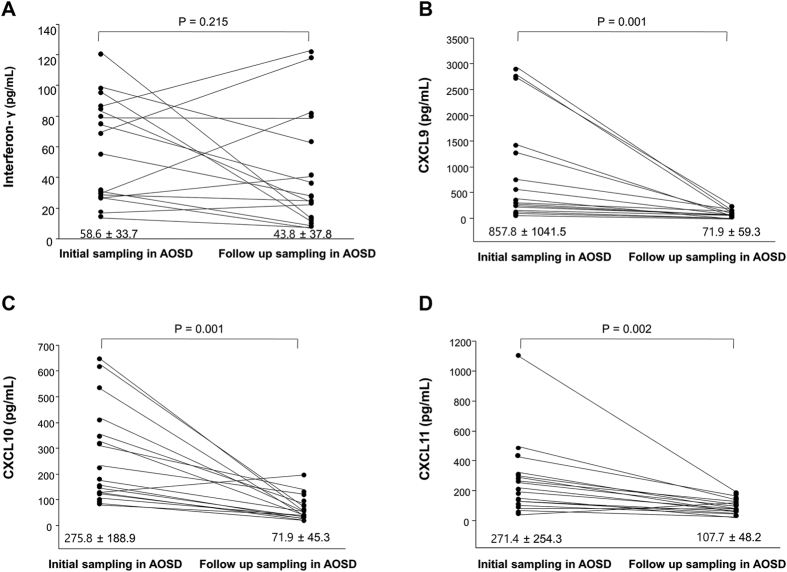
Serum levels of interferon-γ (IFN-γ) (**A**), CXC chemokine 9 (CXCL9) (**B**), CXCL10 (**C**) and CXCL11 (**D**) in follow-up 16 adult-onset Still’s disease (AOSD) patients. Data are expressed as means ± SDs and were analyzed using a Wilcoxon signed-rank test.

Follow-up serum samples were collected from 16 AOSD patients 8.1 ± 6 months after the first samplings. The systemic scores were 1.03 ± 1.06 after treatment with corticosteroid and immunosuppressive agents. The mean levels of IFN-γ, CXCL9, CXCL10 and CXCL11 were 43.8 ± 37.8, 71.9 ± 59.3, 71.9 ± 45.3 and 107.7 ± 48.2 pg/mL, respectively, and their levels significantly decreased compared with initial samples, except for IFN-γ (Fig. 2).

### Serum IFN-α, IFN-β, and TNF-α levels

Although the levels of CXCL9, CXCL10, and CXCL11 were significantly reduced after corticosteroid treatment, the levels of IFN-γ were not reduced. Previous study reported that IFN-α, IFN-β, and TNF-α can induce these chemokines^10^. Therefore, we evaluated serum IFN-α, IFN-β, and TNF-α levels in active and follow-up AOSD patients. However, the levels of IFN-α were not detected in almost AOSD patients except only one (120.9 pg/mL). The mean levels of IFN-β and TNF-α were 105.8 ± 312.0 pg/mL, and 29.7 ± 13.0 pg/mL, respectively, in active AOSD patients. Furthermore, the mean levels of IFN-β and TNF-α were 87.7 ± 130.7 pg/mL, and 21.9 ± 11.0 pg/mL, respectively, in follow-up AOSD patients, and their levels decreased compared with initial samples, but not statistically significant.

### Correlations of serum IFN-γ, CXCL9, CXCL10, and CXCL11 levels with disease manifestations in AOSD patients

Correlations between the levels of disease activity markers and those of serum CXCL9, CXCL10, or CXCL11 in AOSD patients are shown in Table 2. Serum CXCL9 levels correlated with levels of CRP (r = 0.535, *p* < 0.001), ferritin (r = 0.556, *p* < 0.001) and lactate dehydrogenase (LDH) (r = 0.325, *p* = 0.044), as well as systemic scores (r = 0.603, *p* < 0.001). Serum CXCL10 levels correlated with erythrocyte sedimentation rate (ESR) (r = 0.337, *p* = 0.036), CRP (r = 0.543, *p* < 0.001), ferritin (r = 0.513, *p* = 0.001) and LDH (r = 0.364, *p* < 0.023) levels, as well as systemic scores (r = 0.539, *p* < 0.001). Serum CXCL11 levels correlated with CRP (r = 0.509, *p* = 0.001) and ferritin (r = 0.576, *p* < 0.001) levels, and with systemic scores (r = 0.447, *p* = 0.004). Serum CXCL9 levels were also correlated with those of CXCL10 (r = 0.799, *p* < 0.001) and CXCL11 (r = 0.704, *p* < 0.001), and serum CXCL10 levels were correlated with those of CXCL11 (r = 0.706, *p* < 0.001). However, the serum levels of IFN-γ, IFN-β or TNF-α were not correlated with those of CXCR3 ligands.

We also evaluated correlations between changes in CXCL9, CXCL10, or CXCL11 levels and changes in disease activity marker levels in follow-up samples from 16 AOSD patients. Changes in CXCL9 were positively correlated with changes in ferritin (r = 0.715, *p* = 0.002), LDH (r = 0.891, *p* < 0.001) and aspartate transaminase (AST) (r = 0.679, *p* = 0.004) levels, as well as systemic score (r = 0.808, *p* < 0.001). Changes in CXCL10 were correlated with changes in ferritin (r = 0.668, p = 0.005), LDH (r = 0.774, *p* < 0.001) and albumin levels (r = −0.555, *p* = 0.026), as well as systemic score (r = 0.544, *p* = 0.003). Changes in CXCL11 were positively correlated with changes in ferritin (r = 0.515, *p* = 0.041) and LDH (r = 0.588, *p* = 0.017) levels, and systemic score (r = 0.544, *p* = 0.03). Changes in CXCL9 were also correlated with changes in CXCL10 (r = 0.835, *p* < 0.001) and CXCL11 (r = 0.726, *p* = 0.001), and changes in CXCL10 were correlated with changes in CXCL11 (r = 0.665, *p* = 0.005).

An analysis of serum chemokine levels with regard to AOSD manifestations showed that patients with a skin rash had a higher level of CXCL9 (667.6 ± 823.2 vs. 105.8 ± 84.8 pg/mL, *p* = 0.024) and CXCL10 (251.6 ± 191.2 vs. 79.4 ± 45.6 pg/mL, *p* = 0.021) than those without a rash.

### Clinicopathological characteristics of skin

The most prominent skin manifestation was maculopapular eruptions on the upper and lower extremities and the trunk, which affected 25 patients (73.5%). Five patients (14.7%) had persistent pruritic eruptions, two patients (5.9%) had papulopustular lesions on the trunk, and two patients (5.9%) had painful swelling of the low extremities. Most skin biopsies exhibited mild lymphocytic or histiocytic infiltration in the upper dermis. Nuclear debris was evident in the dermis in 20 cases (58.8%). More than half (52.9%) of all cases (18/34) showed interstitial mucin deposition. Macrophage infiltration was evident in 24 cases (70.6%). Nine cases (26.5%) exhibited interface dermatitis with keratinocyte necrosis, and seven (20.6%) exhibited basal vacuolization.

### Immunohistochemical data

**Figure 3:**
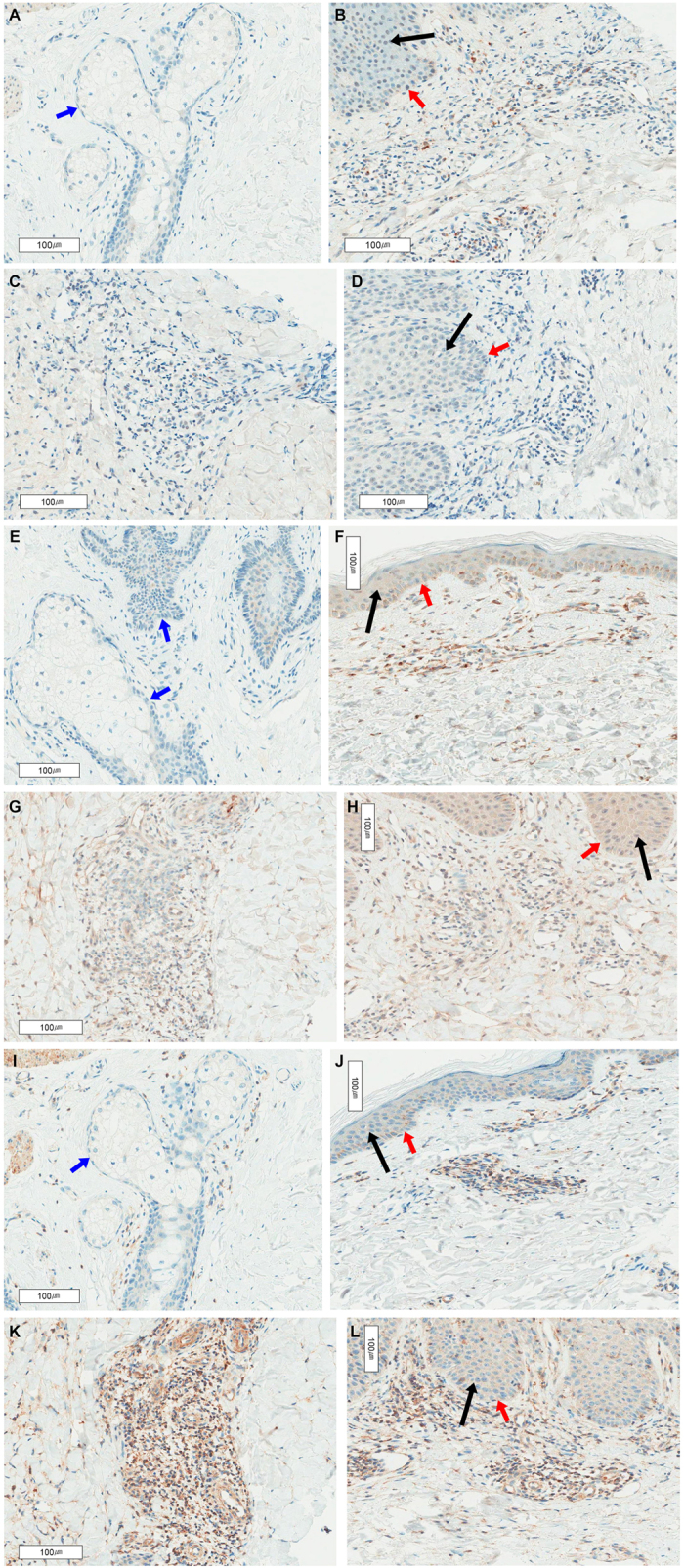
CXC chemokine 9 (CXCL9) (**A**,**B**,**C**,**D**), CXCL10 (**E**,**F**,**G**,**H**) and CXCL11 (**I**,**J**,**K**,**L**) expression in skin biopsies of healthy controls (**A**,**E**,**I**), adult-onset Still’s disease (AOSD) (**B**,**F**,**J**), eczema (**C**,**G**,**K**) and psoriasis (**D**,**H**,**L**) patients. Original magnification, ×200. Note that the percentages of CXCL9- and CXCL10-positive cells are low. However, CXCL9 and CXCL10 were more frequently expressed in the skin of AOSD patients than that of eczematous dermatitis and psoriasis patients. Conversely, CXCL11 was more frequently expressed in the skin of eczematous dermatitis and psoriasis patients than that of AOSD patients. Black arrow: epidermis; red arrow: dermoepidermal junction; blue arrow: pilosebaceous unit.

As a control for CXCL9, CXCL10, CXCL11 and CXCR3 immunohistochemical evaluations, we stained lymphoid cells in the paracortical zone or germinal center of a reactive lymph node. These analyses revealed a granular pattern of cytoplasmic staining. The staining patterns of inflammatory cells in skin biopsies were similar to those of lymphoid cells in a lymph node (Fig. 3). The mean percentages of inflammatory cells expressing CXCL9, CXCL10, CXCL11 and CXCR3 in skin lesions from AOSD patients were 8.7% ± 10.5%, 24.5% ± 21.6%, 27.2% ± 24.3% and 15.2% ± 17.0%, respectively (Table 3). The mean percentages of inflammatory cells expressing CXCL9, CXCL10, CXCL11 and CXCR3 in skin from normal controls were 2.2% ± 2.8%, 4.6% ± 4.9%, 23.8% ± 8.4% and 16.2 ± 24.5%, respectively. In skin lesions from eczema, the mean percentages of inflammatory cells expressing CXCL9, CXCL10, CXCL11 and CXCR3 were 4.8% ± 5.8%, 5.2% ± 6.3%, 63.2% ± 15.7% and 25.2% ± 13.0%, respectively. The mean percentages of inflammatory cells from psoriasis skin lesions expressing CXCL9, CXCL10, CXCL11 and CXCR3 were 3.0% ± 3.4%, 3.0% ± 2%, 46.8% ± 17.7% and 21.2% ± 19.5%, respectively. A comparative analysis showed that the percentage of inflammatory cells expressing CXCL10 in skin lesions from AOSD patients was significantly greater than that in normal controls (*p* = 0.012) and patients with eczema (*p* = 0.019) or psoriasis (*p* = 0.009). By contrast, the percentage of inflammatory cells expressing CXCL11 in AOSD skin lesions was lower than that in eczema (*p* = 0.006) and psoriasis (*p* = 0.035) patients. The percentage of inflammatory cells expressing CXCL9 was only correlated with serum ferritin levels (r = 0.489, *p* = 0.003). Both serum chemokine levels and skin biopsy results were obtained for only five patients. Among these patients, serum CXCL10 levels were correlated with the percentage of inflammatory cells expressing CXCL10 (r = 0.812, *p* = 0.05). Also, 9 of the 34 AOSD patients for which skin biopsy samples were available were concurrently diagnosed with RHS. The percentage of inflammatory cells expressing CXCR3 in AOSD with RHS (30.8% ± 25.1%) was higher than that in AOSD without RHS (9.6% ± 8%, *p* = 0.022). CXCL9-, CXCL10-, CXCL11-, and CXCR3-positive inflammatory cells did not differ in terms of their presence among keratinocyte vacuolization, or karyorrhexis (Table 4). However, the percentage of CXCL9-positive inflammatory cells was higher in patients exhibiting macrophage infiltration than in those who did not (*p* = 0.002). Also, the percentage of CXCL10-positive inflammatory cells was higher in patients exhibiting mucin deposition than in those who did not (*p* = 0.042).

The correlations between CD4, CD8, and CD68 grades and the percentages of CXCL9-, CXCL10-, CXCL11- and CXCR3-positive inflammatory cells in the skin of AOSD patients are shown in Table 5. CXCL10 and CXCR3 levels did not correlate with any grade. However, CXCL9 levels were weakly correlated with grade CD8 (r = 0.411, *p* = 0.016) and CD68 (r = 0.351, *p* = 0.042), and CXCL11 levels were correlated with grade CD4 (r = 0.571, *p* < 0.001).

## Discussion

This study evaluated IFN-γ and the IFN-γ–induced chemokines, CXCL9, CXCL10 and CXCL11, in active, untreated AOSD patients, and compared their levels with those in RA patients and HCs. This is the first study to show that serum CXCL9 and CXCL11 levels are significantly higher in patients with active AOSD than in RA patients and HCs. Furthermore, the serum levels of CXCL9, CXCL10, and CXCL11 in patients with AOSD correlated with those of several inflammatory markers and systemic scores, and their levels fell upon reduction in disease activity in follow-up tests. We also confirmed that these chemokines and their receptor, CXCR3, were expressed in skin rash material from AOSD patients. The percentage of inflammatory cells expressing CXCL10 in AOSD patients was higher than that in normal control, eczema, and psoriasis groups, but the percentage of inflammatory cells expressing CXCL11 in AOSD patients was lower than that in eczema and psoriasis patients.

IFN-γ affects immune responses through immunostimulatory or immunoregulatory actions^19^,^20^. From an immunostimulatory standpoint, IFN-γ promotes proinflammatory responses, such as host defense responses against intracellular pathogens. It also exerts regulatory functions, including inhibition of neutrophil-specific chemokines and induction of T-cell apoptosis. Therefore, in autoimmune or autoinflammatory diseases, IFN-γ could play a disease-reinforcing role or a protective role^20^. This study showed that IFN-γ levels in untreated, acute AOSD patients were significantly higher than those in RA patients and HCs. However, serum IFN-γ levels were not correlated with several AOSD disease activity markers, and were not changed in follow-up sampling. These results are consistent with previous studies^6^,^21^, which also found that serum IFN-γ levels were not different between active and inactive disease states, although serum IFN-γ levels in active AOSD were significantly increased compared with those in HCs^6^.

In addition, we showed markedly higher levels of IFN-γ–induced chemokines in patients with active, untreated AOSD compared with RA patients and HCs, and these chemokines correlated with the levels of several disease activity markers and systemic scores. Furthermore, most follow-up AOSD patients exhibited significantly decreased levels of these chemokine after improvement in disease activity, and changes in these chemokine levels were correlated with changes in levels of disease activity markers and systemic scores. We also found that these chemokines were correlated with each other. Interestingly, serum CXCL9, CXCL10 and CXCL11 were significantly higher in AOSD patients with RHS than in patients without RHS, although the number of RHS patients was small, and serum IFN-γ levels were not different between them. Our results could be interpreted in the same context as previous data related to IL-18 in AOSD^7^,^22^. IL-18, a member of the IL-1 family and a well-known IFN-γ–inducing cytokine, promotes Th1 cytokine production^23^. It has been shown that IL-18 is elevated in the serum, synovial tissue, and lymph nodes of patients with AOSD compared with HCs^7^,^24^. Associations of IL-18 with serum ferritin and CRP levels and neutrophil counts have also been reported^7^. Furthermore, IL-18 levels are significantly elevated in patients with AOSD or systemic JIA complicated by RHS^25^. However, a recent study showed that the levels of IFN-γ and the IFN-γ–induced chemokines, CXCL9, CXCL10 and CXCL11, were not elevated in active systemic JIA without RHS compared with inactive systemic JIA^26^. Moreover, the levels of IFN-γ and IFN-γ–induced chemokines were not associated with laboratory parameters, such as ferritin, CRP and LDH levels in patients with active systemic JIA without RHS. Only the levels of CXCL9, CXCL10, and CXCL11 were weakly correlated with alanine transaminase (ALT) levels. But the data from patients with active systemic JIA with RHS were different. In these patients, the levels of IFN-γ and of IFN-γ–induced chemokines were significantly higher than those in patients with active systemic JIA without RHS. Several disease activity markers, including ferritin and ALT, were also significantly correlated with serum levels of IFN-γ and CXCL9. Although the reason for these differences is not entirely clear, they may be attributable to differences in disease activity of enrolled patients between studies. The patients in our study had an average ferritin level of 7,034.7 ± 11,977.8 ng/mL and ALT level of 66.5 ± 84.2 U/L, but patients with active systemic JIA without RHS had a median ferritin level of 214 ng/mL and an ALT level of 16 U/L. The authors of this previous study also did not compare the levels of these chemokines in systemic JIA patients with those in HCs. In addition, a few patients among active systemic JIA without RHS were treated with some type of medication, whereas all of our patients were untreated prior to sampling.

We further evaluated IFN-γ and IFN-γ–induced chemokine levels according to clinical manifestations of AOSD. These analyses showed that serum CXCL9 and CXCL10 levels were elevated in AOSD patients with an evanescent rash compared with patients who did not display such a manifestation. Subsequent immunohistochemical analyses of skin biopsies for CXCL9, CXCL10 and CXCL11, and their receptor, CXCR3, in 34 patients with active, untreated AOSD compared with normal skin and other inflammatory skin disorders, such as eczema and psoriasis, showed that the three IFN-γ induced chemokines and their receptor, CXCR3, were expressed in inflammatory cells of skin lesions in active AOSD patients. These analyses also revealed some interesting findings. First, the percentage of CXCL10-positive inflammatory cells in the skin of AOSD patients was significantly higher than that in normal skin and skin from eczema and psoriasis patients. We also found enhanced CXCL10 staining in inflammatory cells of skin lesions with mucin deposition, consistent our previous data^9^. However, the frequency of CXCL11-stained cells was significantly lower in AOSD than eczema and psoriasis. Moreover, the intensity of CXCL11 staining was not correlated with that of other chemokines in skin rashes of AOSD patients, although serum levels of CXCL9, CXCL10 and CXCL11 were strongly correlated with each other in acute AOSD patients. Another previous study reported findings similar to our data, showing differences in the expression of CXCL9, CXCL10, and CXCL11 in different types of inflammatory skin disease, including lichen planus, chronic discoid lupus, and psoriasis^27^. The authors of this study suggested that three distinct chemokine expression profiles could be distinguished according to different inflammatory skin diseases, a difference that could be explained by differences in inducers and producer cells^11^. CXCL9 production is induced specifically by IFN-γ, but CXCL10 and CXCL11 can also be induced by IFN-α, IFN-β, and TNF-α^10^. Also, these different ligands for same receptors, CXCR3, can induce different phenotypes. A recent study showed that CXCL11/CXCR3 interaction induces an immunotolerizing condition, although CXCL10/CXCR3 binding drive effector Th1 polarization^28^. Therefore, the expression of IFN-γ–induced chemokines could differ in skin lesions according to disease-specific local conditions, such as redundant inflammatory cells and cytokines. Also, their inflammation condition could be different according redundant chemokine levels. Second, the percentage of CXCR3-positive inflammatory cells in AOSD with RHS was higher than that in AOSD without RHS, a result that contrasts with that obtained for CXCL9, CXCL10, and CXCL11. CXCR3 expression is associated with CD4^+^ Th1, CD8^+^ T cells and natural killer cells, and is consistently expressed by CD4^+^ and CD8^+^ dermal T cells in multiple skin lesions studied^10^,^27^,^29^,^30^. These observations support an important role for IFN-γ–induced chemokines in the recruitment of T-cells into inflammatory skin lesions^27^,^30^. This study further showed that the presence of high CXCR3-expressing inflammatory cells is related to severe systemic conditions, such as RHS.

Because there were no correlations between levels of IFN-γ and those of CXCR3 ligands, we thought of other kinds of cytokine. IFN-α, IFN-β and TNF-α are also known inducer for CXCL10 and CXCL11. Therefore, we evaluated serum IFN-α, IFN-β, and TNF-α levels in active and follow-up AOSD patients. However, these cytokine levels were not correlated with IFN-γ–induced chemokines in AOSD patients. These results suggest that these chemokines could be secreted by other molecules besides these cytokines in the inflammation of AOSD patients. Or these cytokines derived from AOSD patients may have synergistic effect on triggering IFN-γ–induced chemokines, therefore, we could not verify the simplified correlation between each levels. In fact, a recent study with patients of polymyalgia rheumatica showed that serum levels CXCL9 and CXCL10 were elevated although the levels of IFN-γ and TNF-α were not increased^31^.

Among the limitations of this work is the lack of other febrile disorders as a control group for the diagnostic value of these markers. Moreover, we did not compare these markers with chemokine expression in follow-up skin biopsies in AOSD patients. The sample size for subgroup analysis according to disease patterns (monophasic, polycyclic, and chronic articular patterns) and comparison with follow-up samples was also relatively small. In addition, the number of AOSD patients with RHS was small for comparing these levels. Further studies incorporating larger sample sizes, disease activity markers, RHS differentiation markers, and control groups consisting of other febrile disorders are required to fully assess the usefulness of these chemokines in AOSD patients.

In conclusion, we found significantly higher levels of IFN-γ, CXCL9, CXCL10, and CXCL11 in the serum from patients with active AOSD. We confirmed expression of these chemokines and their receptor, CXCR3, in skin rash material from AOSD patients. We also observed that most follow-up samples from AOSD patients showed reduced CXCL9, CXCL10, and CXCL11 levels after improvement in disease activity. These results suggest that IFN-γ–induced chemokines play important roles in the acute systemic inflammatory process and cutaneous inflammation in AOSD. We suggest that serum levels of these chemokines could be useful indicators of disease activity or response to treatment.

## Materials and Methods

### Subjects

Thirty-nine active, untreated AOSD patients, 30 RA patients, and 28 HCs were included in the present study. AOSD patients were diagnosed using Yamaguchi’s criteria after exclusion of individuals with infectious, neoplastic, and autoimmune disorders^32^. In rheumatoid factor-positive patients, a diagnosis of RA was excluded using 1987 American College of Rheumatology (ACR) criteria^33^. In anti-nuclear antibody-positive patients, a diagnosis of systemic lupus erythematosus was excluded using 1982 ACR criteria^34^. Serum samples were collected from all subjects, and follow-up samples after treatment with corticosteroid and immunosuppressive agents were collected from 16 of the 39 AOSD patients 8.1 ± 6 months later. HCs were recruited from among healthy individuals without a medical history of autoimmune, rheumatic, or any other diseases through a public announcement using a screening questionnaire. All blood samples were stored at −70 °C immediately after collection. Information on medical histories, clinical symptoms, and the findings of physical examinations was entered into a database together with serum test results. Each patient underwent a series of laboratory tests, including a complete blood count; ESR evaluation; assessment of CRP, rheumatoid factor, anti-nuclear antibody, LDH and ferritin levels (normal 13–150 ng/mL for females and 30–400 ng/mL for males); liver function testing; and urinalysis. AOSD disease activity was scored as previously described; scores ranged from 0 to 12, with 1 point being given for each of the following manifestations: fever, a typical rash, pleuritis, pneumonia, pericarditis, hepatomegaly or abnormal liver function test data, splenomegaly, lymphadenopathy, leukocytosis ≥ 15,000/mm^2^, sore throat, myalgia, and abdominal pain^4^. This study was approved by the Ajou University Hospital Institutional Review Board (IRB No. AJIRB-BMR-OBS-16-112), and informed consent was obtained from all subjects. All procedures for this study were carried out in accordance with the approved guidelines.

### IFN-γ, CXCL9, CXCL10 and CXCL11 assays

IFN-γ, TNF-α, CXCL9, CXCL10, and CXCL11 levels were measured using commercial enzyme-linked immunosorbent assay (ELISA) kits (R&D Systems, Minneapolis, MN, USA), according to the manufacturer’s instructions. IFN-α and IFN-β were measured using commercial ELISA kits (PBL assay science. Piscataway, NJ, USA).

### Histopathological analysis of skin biopsy samples

Skin biopsies obtained from 34 AOSD patients were sectioned, stained with hematoxylin and eosin, and evaluated as previously described^35^. All slides were independently examined by three pathologists (JHH, JEK, and HY) with respect to the following skin histological parameters: (1) epidermal change, (2) extent of inflammatory cell infiltration, and the presence of (3) karyorrhexis, (4) vasculitis, and (5) interstitial mucin.

### Immunohistochemical evaluation

We previously evaluated CXCL10 and CXCR3 in 26 AOSD patients, and reported those findings^9^. In the current study, we recruited an additional eight untreated, active AOSD patients, for a total of 34 AOSD patients. For comparative analyses, skin biopsy samples were obtained from five normal, five psoriasis, and five eczematous dermatitis patients. Immunohistochemistry was performed on formalin-fixed, paraffin-embedded sections using a Benchmark XT automated staining system (Ventana Medical Systems Inc., Tucson, AZ, USA). The primary antibodies used were those against CD4 (1:10 dilution) and CD8 (1:50 dilution), from Thermo Fisher Scientific (Fremont, CA, USA); CD68 (1:200 dilution), from Novocastra Laboratories Ltd (Newcastle, United Kingdom); and CXCL9 (1:50 dilution), CXCL10 (1:50 dilution), CXCL11 (1:50 dilution) and CXCR3 (1:20 dilution), from R&D Systems. Immunoreactive proteins were detected using a Ventana Optiview DAB kit (Ventana Medical Systems). Scores were calculated by dividing the numbers of positive inflammatory cells by the total number of inflammatory cells, expressed as a percentage (CXCL9, CXCL10, CXCL11, and CXCR3) or graded on the following 1 to 3 scale (CD4, CD8, and CD68): 1, 1–33%; 2, 34–66%; and 3, 67–100%.

### Statistical analyses

All data are presented as means ± standard deviations. Differences in CXCL9, CXCL10, and CXCL11 levels between groups were evaluated using an independent t-test or the Mann-Whitney U-test. Correlations between histological scores and disease activity marker levels were evaluated using Pearson’s correlation test or Spearman’s correlation test. The Wilcoxon signed-rank test was also used to compare IFN-γ, CXCL9, CXCL10, and CXCL11 levels in patients who underwent follow-up serum sampling. All statistical analyses were performed using SPSS version 23.0 (SPSS, Chicago, IL, USA). A p-value < 0.05 was regarded as indicative of statistical significance.

## Additional Information

**How to cite this article**: Han, J. H. *et al*. Elevated circulating levels of the interferon-γ–induced chemokines are associated with disease activity and cutaneous manifestations in adult-onset Still’s disease. *Sci. Rep.* **7**, 46652; doi: 10.1038/srep46652 (2017).

**Publisher's note:** Springer Nature remains neutral with regard to jurisdictional claims in published maps and institutional affiliations.

## Figures and Tables

**Table 1:** Clinical characteristics of patients.

	AOSD (n = 39)	RA (n = 30)	HC (n = 28)
Age (years)	42.9 ± 16.8	48.7 ± 9.6	42.1 ± 10.8
Gender (F/M)	33/6	26/4	25/3
Fever	37 (94.9)		
Sore throat	22 (56.4)		
Skin rash	34 (87.2)		
Lymphadenopathy	14 (35.9)		
Splenomegaly	8 (20.5)		
Hepatomegaly	8 (20.5)		
Pericarditis	6 (15.4)		
Pleuritis	5 (12.8)		
Arthralgia	36 (92.3)		
Arthritis	21 (53.8)		
Hemoglobin, g/dL	11.1 ± 1.7	12.8 ± 1.5	
Leukocyte,/μL	13,379 ± 4,720	7,783 ± 3,348	
Platelet, ×10^3^/μL	311.1 ± 113.2	256.7 ± 66.4	
Ferritin, ng/mL	7,034.7 ± 11,977.8		
LDH, U/L	466.8 ± 429.7		
Inteferon-β, pg/mL	105.8 ± 312.0		
TNF-α, pg/mL	29.7 ± 13.0		
ESR, mm/h	56.3 ± 20.1	31.1 ± 23.7	
CRP, mg/dL	7.73 ± 5.4	1.15 ± 2.49	
AST, U/L	73.9 ± 63.9	24.7 ± 10.4	
ALT, U/L	66.5 ± 84.2	23.5 ± 18	
Bilirubin, mg/dL	0.59 ± 0.64	0.57 ± 0.21	
Albumin, g/dL	3.71 ± 0.64	4.42 ± 0.3	
ANA positivity	4 (10.3)	7 (23.3)	
RF positivity	2 (5.2)	27 (90)	
Systemic score	5.38 ± 1.33		
DAS-28		3.95 ± 1.22	

AOSD, adult onset Still’s disease; RA, rheumatoid arthritis; HC, healthy controls; LDH, lactate dehydrogenase; TNF, tumor necrosis factor; ESR, erythrocyte sedimentation rate; CRP, C-reactive protein; AST, aspartate transaminase; ALT, alanine transaminase; ANA, antinuclear antibody; RF, rheumatoid factor. DAS-28, disease-activity score that includes 28 joints. All values are presented as numbers (with percentages) or means ± SD. The systemic scoring system of Pouchot *et al*.^4^ assigns a score from 0 to 12, with 1 point for each of the following manifestations: fever, typical rash, pleuritis, pneumonia, pericarditis, hepatomegaly or abnormal liver function test data, splenomegaly, lymphadenopathy, leukocytosis ≥ 15,000/mm^2^, sore throat, myalgia, and abdominal pain.

**Table 2:** Correlations of CXCL9, CXCL10, and CXCL11 levels with disease activity markers and between each other in 39 AOSD patients and correlations between changes (Δ) in serum CXCL9, CXCL10 or CXCL11 levels and those of disease activity markers in follow-up 16 AOSD samples.

Disease activity marker	Correlation coefficient, r (*p*-value)					
	CXCL9	CXCL10	CXCL11	ΔCXCL9	ΔCXCL10	ΔCXCL11
(Δ) Systemic score	**0.603** (<**0.001**)	**0.539** (<**0.001**)	**0.447** (**0.004**)	**0.808** (<**0.001**)	**0.541** (**0.031**)	**0.544** (**0.03**)
(Δ) Leukocyte	0.06 (0.718)	0.036 (0.827)	0.103 (0.534)	0.487 (0.056)	0.252 (0.347)	0.386 (0.14)
(Δ) Hemoglobin	−0.031 (0.849)	0.046 (0.78)	−0.041 (0.803)	−0.091 (0.737)	−0.146 (0.59)	0.068 (0.803)
(Δ) Platelet	−0.164 (0.317)	−0.169 (0.303)	0.098 (0.551)	−0.188 (0.485)	−0.335 (0.204)	−0.306 (0.249)
(Δ) ESR	0.264 (0.105)	0.337 (0.036)	0.108 (0.512)	0.447 (0.083)	0.238 (0.374)	0.138 (0.61)
(Δ) CRP	**0.535** (<**0.001**)	**0.543** (<**0.001**)	**0.509** (**0.001**)	0.368 (0.161)	0.294 (0.269)	0.121 (0.656)
(Δ) Ferritin	**0.556** (<**0.001**)	**0.513** (**0.001**)	**0.576** (<**0.001**)	**0.715** (**0.002**)	**0.668** (**0.005**)	**0.515** (**0.041**)
(Δ) LDH	**0.325** (**0.044**)	**0.364** (**0.023**)	0.145 (0.378)	**0.891** (<**0.001**)	**0.774** (<**0.001**)	**0.588** (**0.017**)
(Δ) Albumin	−0.033 (0.841)	0.019 (0.909)	−0.052 (0.755)	−0.386 (0.139)	−**0.555** (**0.026**)	−0.310 (0.243)
(Δ) Bilirubin	0.128 (0.438)	0.024 (0.887)	0.063 (0.704)	−0.136 (0.616)	0.016 (0.952)	0.019 (0.944)
(Δ) AST	0.084 (0.611)	0.072 (0.664)	0.093 (0.575)	**0.679** (**0.004**)	0.474 (0.064)	0.424 (0.102)
(Δ) ALT	−0.102 (0.535)	−0.18 (0.272)	−0.078 (0.638)	0.071 (0.765)	−0.127 (0.64)	−0.234 (0.383)
(Δ) IFN-γ	0.201 (0.219)	0.023 (0.888)	0.223 (0.172)	−0.171 (0.528)	−0.156 (0.564)	−0.044 (0.871)
(Δ) IFN-β	−0.091 (0.647)	0.069 (0.728)	−0.048 (0.810)	−0.213 (0.464)	−0.059 (0.840)	−0.275 (0.342)
(Δ) TNF-α	0.263 (0.176)	0.199 (0.309)	0.213 (0.276)	0.099 (0.737)	0.068 (0.817)	−0.002 (0.994)
(Δ) CXCL10	**0.799** (<**0.001**)			**0.835** (<**0.001**)		
(Δ) CXCL11	**0.704** (<**0.001**)	**0.706** (<**0.001**)		**0.726** (**0.001**)	**0.665** (**0.005**)	

ESR, erythrocyte sedimentation rate; CRP, C-reactive protein; LDH, lactate dehydrogenase; AST, aspartate transaminase; ALT, alanine transaminase; IFN-γ, interferon-γ; TNF-α, tumor necrosis factor-α. Pearson’s correlation coefficients were calculated. The systemic scoring system of Pouchot *et al*.^4^ assigns a score from 0 to 12, with 1 point for each of the following manifestations: fever, typical rash, pleuritis, pneumonia, pericarditis, hepatomegaly or abnormal liver function test data, splenomegaly, lymphadenopathy, leukocytosis ≥ 15,000/mm^2^, sore throat, myalgia, and abdominal pain.

**Table 3:** C-X-C motif chemokine 9 (CXCL9), CXCL10, CXCL11 and C-X-C motif receptor-3 (CXCR3) immunostaining results in skin biopsies from 34 adult-onset Still’s disease (AOSD) patients, 5 healthy controls (HC), 5 eczema patients, and 5 psoriasis patients.

	Staining cell percent AOSD	Staining cell percent normal skin	*P*-value (AOSD vs. normal)	Staining cell percent eczema	*P*-value (AOSD vs. eczema)	Staining cell percent psoriasis	*P*-value (AOSD vs. psoriasis)
CXCL9	8.7 ± 10.5	2.2 ± 2.8	0.098	4.8 ± 5.8	0.581	3.0 ± 3.4	0.178
CXCL10	**24.5 ± 21.6**	**4.6 ± 4.9**	**0.012**	**5.2 ± 6.3**	**0.019**	**3.0 ± 2.0**	**0.009**
CXCL11	**27.2 ± 24.3**	23.8 ± 8.4	0.474	**63.2 ± 15.7**	**0.006**	**46.8 ± 17.7**	**0.035**
CXCR3	15.2 ± 17.0	16.2 ± 24.5	0.791	25.2 ± 13.0	0.074	21.2 ± 19.5	0.257

*P*-values determined using a Mann-Whitney U-test.

**Table 4:** Percentages of inflammatory cell staining for various chemokines according to pathologic findings.

Pathologic findings	(+), n	(−), n	P value	Pathologic findings	(+), n	(−), n	P value
Keratinocyte vacuolization	N = 7	N = 27		Macrophage infiltration	N = 24	N = 10	
CXCL9	10.3 ± 12.7	8.3 ± 10.1	0.708	CXCL9	11.58 ± 11.3	1.8 ± 1.6	0.002
CXCL10	31.3 ± 24.8	22.7 ± 20.8	0.427	CXCL10	26.8 ± 18.9	18.9 ± 14	0.589
CXCL11	28.3 ± 23.4	22.9 ± 29.3	0.357	CXCL11	26.2 ± 25.4	29.6 ± 22.6	0.642
CXCR3	21.1 ± 22.7	13.6 ± 15.4	0.835	CXCR3	14.7 ± 16.3	16.3 ± 19.6	0.926
Parakeratosis	N = 6	N = 28		Karrhyorrhexis	N = 20	N = 14	
CXCL9	9.7 ± 5.5	8.5 ± 11.4	0.413	CXCL9	11 ± 12.3	5.5 ± 6.2	0.204
CXCL10	32 ± 19.7	22.9 ± 22	0.257	CXCL10	26.5 ± 26.1	21.6 ± 13.2	1
CXCL11	47.8 ± 35.9	22.8 ± 19.2	0.204	CXCL11	27.7 ± 25.4	26.4 ± 23.7	1
CXCR3	17.8 ± 14.2	14.6 ± 17.7	0.466	CXCR3	17.4 ± 17.1	12.1 ± 17.1	0.077
Keratinocyte necrosis	N = 9	N = 25		Mucin	N = 18	N = 16	
CXCL9	12.3 ± 13.3	7.4 ± 9.3	0.13	CXCL9	9.1 ± 11.4	8.3 ± 9.7	0.905
CXCL10	21.3 ± 23.1	25.6 ± 21.4	0.489	CXCL10	32.1 ± 25.1	16 ± 12.9	0.042
CXCL11	17.4 ± 16.5	30.7 ± 26	0.13	CXCL11	35.8 ± 28.5	17.5 ± 13.9	0.055
CXCR3	17.8 ± 20.4	14.2 ± 16	0.514	CXCR3	15.7 ± 14.5	14.6 ± 20	0.365

All values are means ± SD. CXCL9, C-X-C motif chemokine 9; CXCR3, C-X-C motif receptor-3.

**Table 5:** Correlations between inflammatory cell grade (CD4/CD8/CD68 staining) and the percentages of inflammatory cells staining for CXCL9, CXCL10, CXCL11, and CXCR3.

IHC	Correlation coefficient, r (*p*-value)			
	CXCL9	CXCL10	CXCL11	CXCR3
CD4	0.062 (0.727)	−0.077 (0.667)	**0.571** (<**0.001**)	−0.051 (0.775)
CD8	**0.411** (**0.016**)	0.191 (0.278)	−0.203 (0.25)	0.289 (0.098)
CD68	**0.351** (**0.042**)	0.155 (0.381)	0.129 (0.466)	−0.039 (0.826)

Differences were evaluated by calculating Spearman’s correlations. IHC, immunohistochemical stain; CXCL9, C-X-C motif chemokine 9; CXCR3, C-X-C motif receptor-3; CD4, cluster of differentiation 4.
